# Supplementary figures and images for: High Risk α-HPV E6 Impairs Translesion Synthesis by Blocking POLη Induction
Source: Cancers (Basel). 2020 Dec 23;13(1):28. doi: 10.3390/cancers13010028 (PMC7793514; doi:10.3390/cancers13010028)

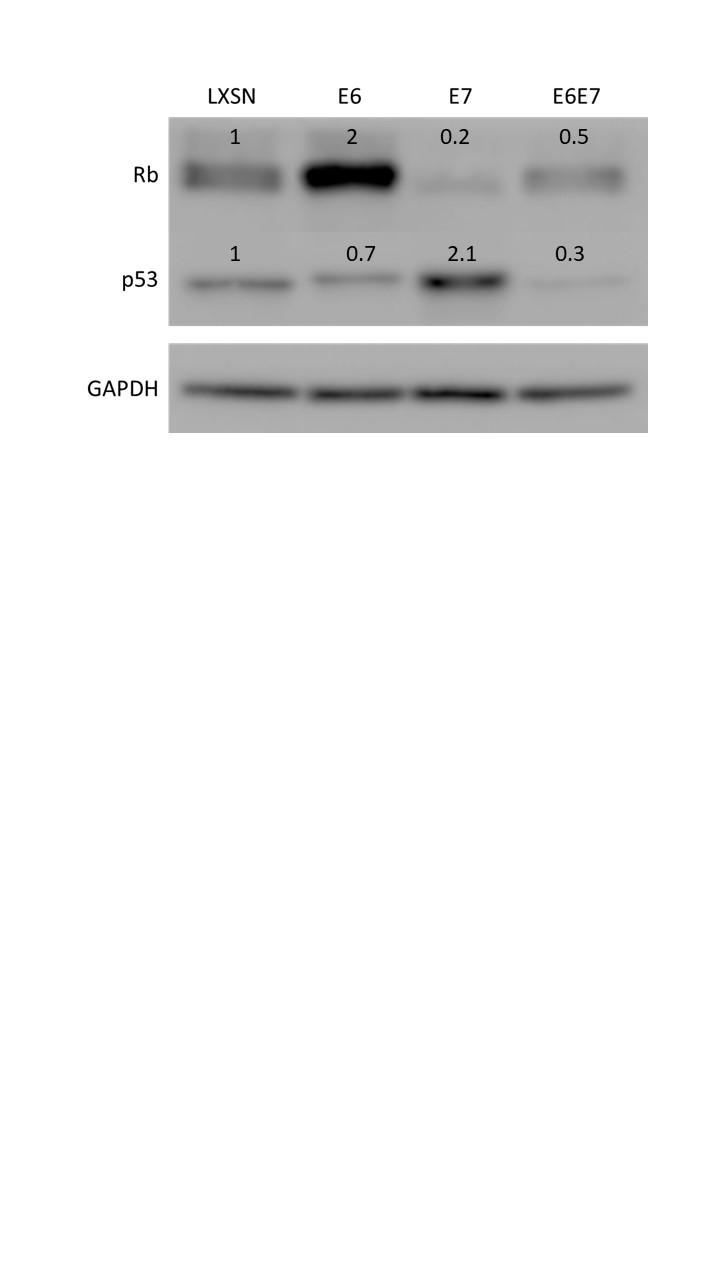

Supplement: Supplementary file 1 [file cancers-13-00028-s001.zip › Figure S1.jpg]
